# Solidification and Segregation Behaviors of Superalloy IN718 at a Slow Cooling Rate

**DOI:** 10.3390/ma11122398

**Published:** 2018-11-28

**Authors:** Xiao Shi, Shengchao Duan, Wensheng Yang, Hanjie Guo, Jing Guo

**Affiliations:** 1 School of Metallurgical and Ecological Engineering, University of Science and Technology Beijing, Beijing 100083, China; mighty_works@163.com (X.S.); metall_dsc@163.com (S.D.); yws901125@163.com (W.Y.); guojing@ustb.edu.cn (J.G.); 2 Beijing Key Laboratory of Special Melting and Preparation of High-End Metal Materials, Beijing 100083, China

**Keywords:** INCONEL 718, solidification, segregation, slow cooling rate

## Abstract

The solidification and micro- and macro-segregation behaviors of as-cast INCONEL 718 (IN718) alloy at different temperatures under a slow cooling rate (5 °C/min) were investigated in this study. The results indicate that the solid-liquid interface grows into reticulation of hexagons during solidification. The variation trend of the solid fraction and transition rate of the solid phase with solidification time can be well characterized by the Boltzmann and Gaussian distribution, respectively. The order of segregation degree of negative segregation elements is: Fe > Cr > Al. Nb is the most principal positive segregation element, which is abundant in the long-term unsolidified remaining liquid. At the terminal stage of solidification, the increasing tendencies of the Nb and Mo contents in the liquid and the residual liquid density with decreasing temperature reverse due to the formation of the Laves phase. The freckles are most likely to form in the early stages of solidification, at which the liquid fraction is between 0.3 and 0.2, and the temperature range is about 1320 °C to 1310 °C. The information produced is expected to characterize the solidification and segregation behaviors of IN718 alloy when cooled at a slow rate characteristic of larger ingots typical of those required for industrial gas turbines and aircraft engines.

## 1. Introduction

Nickel-based superalloy, INCONEL 718 (IN718), is widely used as raw materials to manufacture components for the aerospace, nuclear, and land-based turbine industry [1,2]. It is a precipitation strengthened alloy containing a large amount of strengthening elements, such as Nb, Mo, Al, Ti, and Cr, which can prominently improve the corrosion resistance, weldability, creep strength, rupture strength, and high temperature structural stability of the alloy in the temperature range of −253 °C to 650 °C [3,4,5]. These properties depend to a large extent on the microstructure produced by liquid metal processing [6]. In such process of liquid metal cooling, the actual cooling rate is greater than that of the equilibrium in most cases. The solute redistribution will thus occur significantly due to the high content of additional alloying elements, resulting in the formation of a non-uniform matrix and harmful segregation constituents [5,7]. Consequently, some solidification defects, such as element segregation and brittle intermetallic compounds, randomly form during solidification [8], which leads to considerable difficulties in subsequent processing steps and a significant deterioration in the performance of the final castings.

Many researchers have provided a variety of valuable information to characterize or predict the solidification and segregation behaviors of IN718 superalloy during casting or welding [8,9,10,11,12,13,14]. It is known that two eutectic-type reactions, *L*→*γ*+NbC and *L*→*γ*+Laves, will inevitably occur during the non-equilibrium solidification of alloy IN718. The NbC carbide and Laves phase, which are considered to be the main microstructural segregation constituents, will be precipitated in the solute-rich interdendritic liquids at different stages of solidification. Their formations depend on the local non-equilibrium conditions of solidification and corresponding degree of segregation of elements [15]. With the development of aircraft, steam turbine, and other industries, higher requirements are set on larger sized IN718 ingots and castings. The scale-up in the diameter inevitably leads to a reduction in the cooling rate inside the ingot, which will greatly increase the inhomogeneity of the chemical composition [12]. Therefore, the solidification characteristics at slow cooling rates may be different from the general ones due to the change of local solidification conditions; thus, it is meaningful to further study the microstructures and micro-segregation of IN718 alloy to determine whether new information can be found under slow-cooling conditions.

Furthermore, large diameter nickel-based superalloy castings and ingots are widely accepted to have a marked tendency to form macro-segregation defects, such as freckles [16,17,18,19], which appear as long trails of equiaxed grains with compositions and microstructures different from the bulk alloy and constitute crack initiation sites, which cannot be eliminated by homogenization or thermomechanical treatments [20]. Freckles are highly undesirable in critical applications because they have a considerable adverse effect on mechanical performance [21]. Castings and ingots containing freckles must be scrapped [16,21]. Therefore, understanding the formation trend of freckles is a major key toward the successful manufacture of large superalloy ingots [21]; thus, it is essential to evaluate quantitative insight on the freckling conditions at slow cooling rates.

Based on the above discussion, it is essential to continue efforts for a better understanding of the solidification features of IN718 alloy under slow-cooling conditions to expand the processing flow and further increase the size of castings and ingots to meet the requirements for the new generation of land-based turbines and large aircraft engines. The purpose of this work is to conduct fundamental research on the solidification and micro- and macro-segregation behaviors of IN718 alloy under a slow cooling rate in the laboratory, and to provide basic experimental data and forecasts to guide the control of micro- and macro-structures in the casting applications.

## 2. Experimental Procedures 

### 2.1. Experimental Material and Compositions

The IN718 samples used in this study were extracted from the center of a 21 kg ingot produced by vacuum-induction melting (VIM). The chemical compositions of the VIM ingot and the AMS specification (AMS 5596A) [22] for IN718 are presented in Table 1.

### 2.2. In Situ Observation

To directly observe the evolution of the solid-liquid interface during solidification and to accurately investigate the microstructural characteristics at different temperatures, the solidification experiments combined with the in situ observation method were performed on a confocal laser scanning microscopy (CLSM), which can provide advantageous real-time and continuous observations of the solidification process on the sample surface at elevated temperatures up to 1600 °C. The IN718 specimens, which were ground and mirror polished into thin discs with a diameter of 7.6 mm and a height of 2.5 mm, were in turn inserted into the chamber of the CLSM (VL2000DX-SVF17SP, LASERTEC Inc., Yokohama, Japan) after being separately placed into high-purity alumina crucibles. After the gases in the chamber were evacuated, the chamber was continuously filled with ultra-pure argon gas (≥99.999 %) to prevent the oxidation of the sample surface. It should be noted that the solidification of a sample has commenced below the surface of the opaque melt and is gradually being revealed on the melt surface because the thermocouple is in contact with the bottom of the alumina crucible [8,23]. Therefore, the temperature difference between the surface of the sample and thermocouple should be taken into account in the experiment.

Given that the general range of cooling rates for industrial ingots is basically less than 20 °C/min [24], and based on our previous studies, the lower the cooling rate, the stronger the segregation tendency of Nb and Mo during solidification [7]. In this continuous quenching experiment, the cooling rate is set to the minimum cooling rate that can be accurately measured by the experimental equipment, that is, 5 °C/min. Each sample was heated from room temperature to 1450 °C in the CLSM chamber and held for 3 min to homogenize the liquid phase. When the liquid samples were then cooled from 1450 °C at a rate of 5 °C/min, the solidification processes were interrupted by quenching with helium gas (the cooling rate was approximately 6000 °C/min at this stage) at selected temperatures to preserve the microstructures of the samples at high temperatures. According to the results of differential scanning calorimetry (DSC; STA-449C-Jupiter, NETZSCH Inc., Selb, Germany) measurements of the same material at the cooling rate of 5 °C/min in this study, the quenching temperatures were designed as 1320, 1310, 1300, 1270, 1250, 1190, 1170, and 1100 °C (completely solidified), respectively.

### 2.3. Element Determination and Microstructural Analysis

Each solidified sample after in situ observation via CLSM was mechanically polished and electrolytically etched in a mixture solution of CrO_3_ (15 g) + H_2_SO_4_ (10 mL) + H_3_PO_4_ (150 mL) at a voltage of 10 V for 10 s. The electron-probe microanalysis (EPMA; 1720H, SHIMADZU Inc., Kyoto, Japan), in backscattered electron mode, was used to determine the elements qualitatively and quantitatively in different regions. The microstructures were analyzed by field-emission scanning electron microscopy (FESEM; SUPRA 55, ZEISS Inc., Oberkochen, Germany) in combination with attached energy dispersive X-ray spectroscopy (EDS). The secondary dendrite arm spacing in the completely solidified microstructure was measured through multiple optical images using the linear intercept method.

## 3. Results and Discussion

### 3.1. Solidification Behavior

#### 3.1.1. DSC Measurement

**Figure 1:**
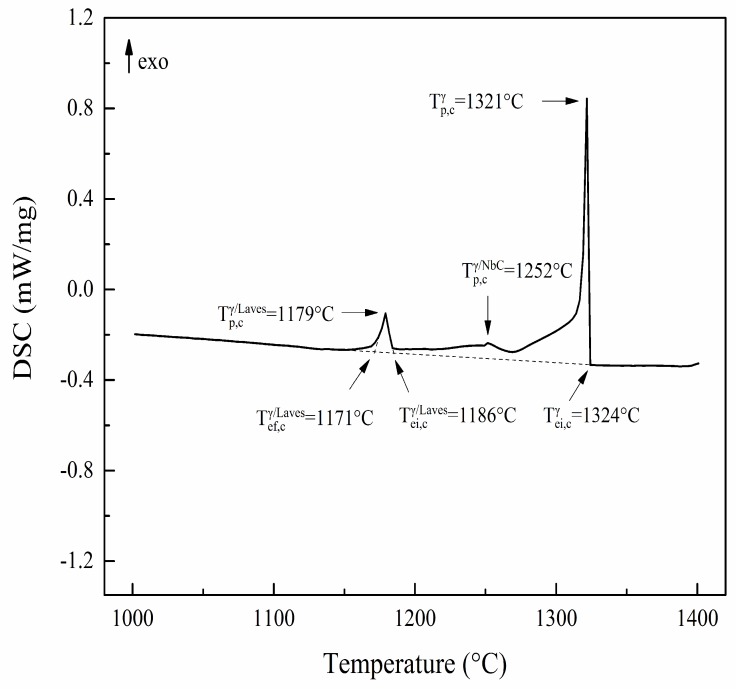
DSC curve and reaction temperatures of experimental IN718 specimen.

The main purpose of the DSC measurement (5 °C/min, cooling) in this work is to identify the phase transformation temperatures of the studied IN718 alloy during solidification and to set the temperature parameters for subsequent CLSM continuous quenching experiments. The DSC result is shown in Figure 1. “T_ei,c_” is the extrapolated initial cooling temperature, representing that a phase just begins to solidify in the process of cooling. “T_ef,c_” is the extrapolated end cooling temperature, denoting that a phase has just been solidified. “T_p,c_” is the temperature of the exothermic peak. The superscripts indicate the reaction phase within each temperature range. A total of three exothermic peaks can be clearly observed during the cooling process of 5 °C/min. When the temperature decreases to about 1324 °C, the initial *γ* solid solution first nucleates and grows in the liquid phase, releasing large amounts of latent heat of crystallization, resulting in a distinct exothermic peak in the curve. A slight exothermic peak is then observed in the vicinity of 1252 °C, at which stage the reaction of *L*→*γ*+NbC can be considered to occur. Finally, a terminal solidification reaction appears as the temperature is below 1186 °C, where the eutectic reaction of *L*→*γ*+Laves is reached. With continued cooling to 1171 °C, the sample is solidified completely and the DSC curve returns to the baseline. The above interpretations of the DSC curve are consistent with the previous investigations of differential thermal analysis (DTA) profiles by Knorovsky et al. [15] and Antonsson et al. [22].

According to the measurement results of DSC, the solidification process of IN718 alloy can be roughly divided into three stages: *L*→*γ*, *L*→*γ*+NbC, and *L*→*γ*+Laves. The phase transformation behaviors and microstructure evolution of the alloy during solidification can thus be specifically analyzed by quenched samples in each temperature range.

#### 3.1.2. CLSM Observations

**Figure 2:**
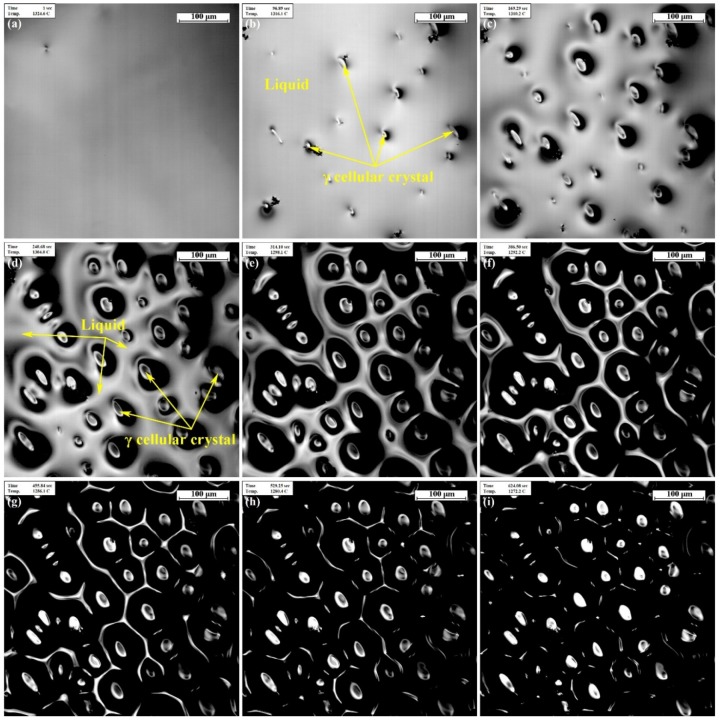
In situ observation results of the solidification process at different temperatures: (**a**) 1.00 s, 1324.6 °C; (**b**) 96.89 s, 1316.1 °C; (**c**) 169.29 s,1310.2 °C; (**d**) 240.68 s, 1304.0 °C; (**e**) 314.10 s, 1298.1 °C; (**f**) 386.50 s, 1292.2 °C; (**g**) 455.84 s, 1286.1 °C; (**h**) 529.25 s, 1280.4 °C; (**i**) 624.08 s, 1272.2 °C. The scale bars in the figures are 100 μm.

Figure 2 shows the recorded typical CLSM in situ micrographs of nucleation and growth of the primary *γ* phase in the liquid alloy during the solidification process with a cooling rate of 5 °C/min. The *γ* solid phase first appears on the liquid surface when the temperature is around 1325 °C. With further cooling to 1300 °C, many nuclei generate and grow rapidly to form a cellular interface. As the temperature decreases further, the cellular structures grow into hexagonal-cell crystals. Additionally, the liquid area continuously decreases and forms a certain number of continuous reticular intercellular regions, which does not completely solidify over the next wide temperature range. It can be inferred that the intercellular fluid areas have a lower melting point than the solid portion, which may be due to a large amount of solutes being discharged from the solidifying front to the residual liquid in the course of solidification. Consequently, the most severe element segregation certainly occurs in such intercellular grooves, where the NbC and Laves phase are most likely to form in the subsequent solidification process. Unfortunately, the undulating cellular interface adversely affects the depth of field of the microscope, which exceeds the present equipment capability, so the changes in micromorphology of the sample surface cannot be observed clearly below 1250 °C through CLSM.

**Figure 3:**
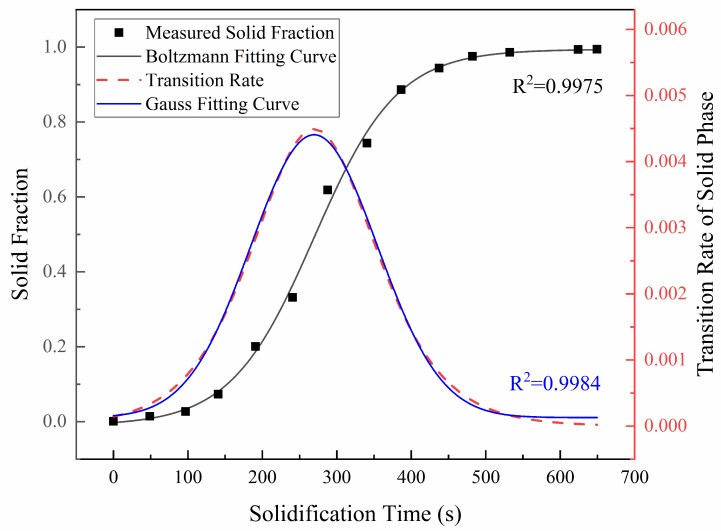
The variation trend of the solid fraction and the transition rate of the solid phase with solidification time.

The solid fractions on the free surface at different solidification times were measured through multiple grayscale images processed by in situ micrographs using the Image-Pro Plus software. The results are presented in Figure 3, indicating that the growth of the primary *γ* phase in the liquid alloy can also be divided into three stages: The initial transient stage, the rapid growth stage, and the late slow solidification stage. This trend can be well characterized by the Boltzmann distribution, and the fitting relationship between the solid fraction (*f*_s_) and the solidification time (*t,s*) under 5 °C/min cooling rate in this study is established as:

(1) $$f_{s}=1-1/(1+\exp((t-269.5)/55.9))$$

The transition rate of the solid phase can be reflected by the differential of the fitting curve of the solid fraction with respect to solidification time. The calculated result is superimposed in Figure 3, which reveals that the solid phase transition rate rapidly increases to a peak value at the rapid growth stage of solidification and then begins to decrease, and always approaches nearly zero in the late slow solidification stage. Furthermore, the relationship between the transition rate of the solid phase ($\frac{df_{s}}{dt}$) and the solidification time (*t,s*) also exhibits a feature of Gaussian distribution, which gives a following functional form in the cooling conditions studied here:

(2) $$\frac{df_{s}}{dt}=1.3\times 10^{-4}+4.3\times 10^{-3}\times \exp(-2\times \left(\frac{t-269.5}{170.2}\right){}^{2})$$

### 3.2. Microsegregation Analysis

**Figure 4:**
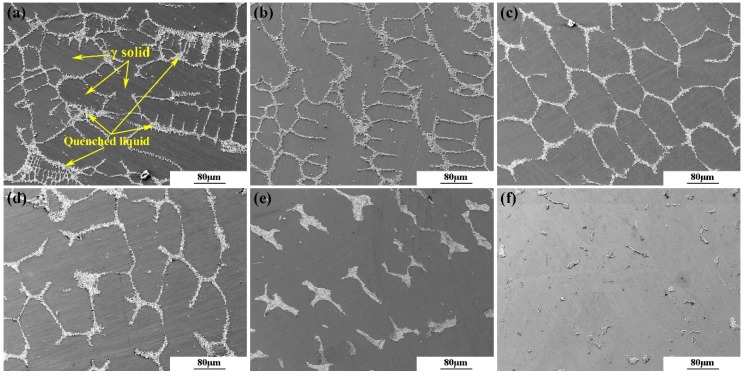
BSE images of the samples at different quenching temperatures: (**a**) 1320 °C; (**b**) 1300 °C; (**c**) 1270 °C; (**d**) 1250 °C; (**e**) 1190 °C; (**f**) 1170 °C.

Figure 4 shows backscattered electron (BSE) images of each quenched sample. It can be seen from the figure that as the temperature decreases, the area of liquid phase decreases, and these reticular liquid regions become increasingly dispersed and discontinuous. The average solid fraction of the sample at each quenching temperature was also evaluated, and the results are listed in Table 2. When the liquid alloy is cooled to 1320 °C, the solid fraction has reached 71%, whereas the remaining liquid does not completely disappear in the next wide temperature range until the temperature is below 1170 °C.

**Figure 5:**
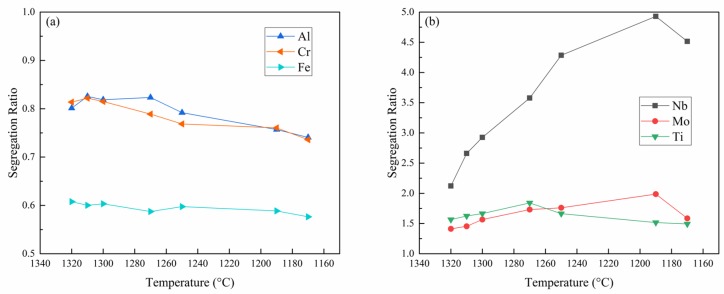
Segregation ratios of solutes at different temperatures: (**a**) Al, Cr, and Fe; (**b**) Ti, Nb, and Mo.

The average contents of all the major alloying elements of IN718 alloy, including Nb, Mo, Al, Ti, Cr, and Fe, in the quenched liquid of each sample were quantitatively measured by EPMA, respectively, the results are also listed in Table 2. Then, the segregation ratio (SR), which is determined by dividing the content of solute in the liquid by the nominal composition [14], can be obtained, as shown in Figure 5. The variation trend of SR could reflect an extent of segregation effects [25]. It can be clearly seen from Figure 5 that the SRs of Cr, Fe, and Al are always less than 1 during solidification, which means that they are negative segregation elements that are plentifully enriched in the dendrite arms. Whereas the SRs of Ti, Nb, and Mo are greater than 1 all the time, indicating that they are positive segregation elements that tend to be rejected into the interdendritic residual liquid. The larger the deviation between SR and 1, the greater the extent of the segregation effects. Therefore, among the negative segregation elements, the segregation degree of Fe is greater than those of Al and Cr, and Cr is slightly more segregated compared with Al; thus, the order of their degree of segregation is: Fe > Cr > Al. On the whole, the degree of segregation of these negative segregation elements follows a slight increasing trend with decreasing temperature in the course of solidification. Among the positive segregation elements, Nb shows the strongest tendency to segregate into the liquid phase. The SR of Ti shows a tendency to increase first and then decrease. However, for Nb and Mo, a lower temperature results in a greater degree of segregation, with the exception of the 1170 °C quenched sample, in which the SRs of Nb and Mo are manifestly milder than those in the former specimen.

**Figure 6:**
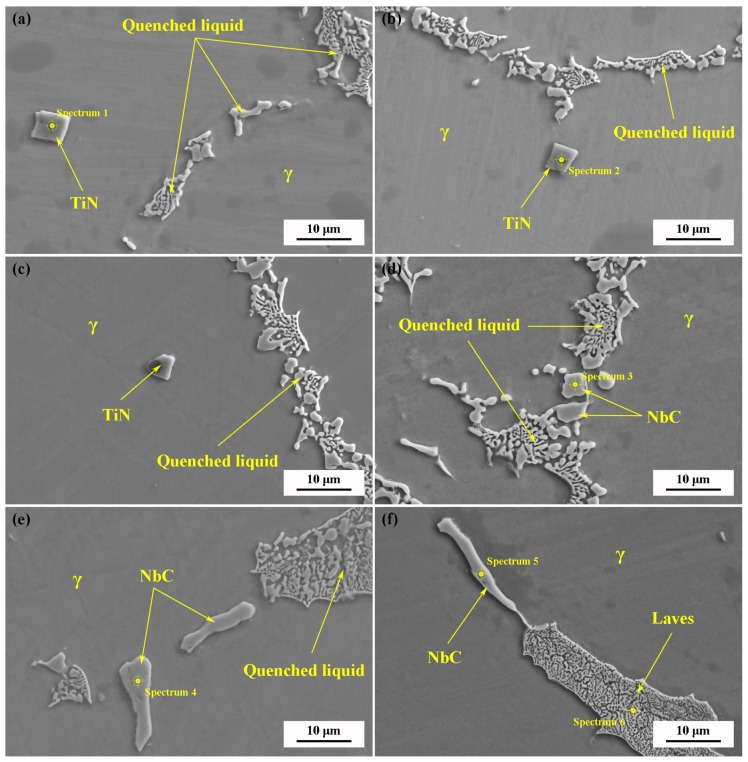
FESEM images of the samples at different quenching temperatures: (**a**) 1320 °C; (**b**) 1300 °C; (**c**) 1270 °C; (**d**) 1250 °C; (**e**) 1190 °C; (**f**) 1170 °C.

Figure 6 and Table 3 present FESEM images and EDS analysis of the samples quenched at different temperatures. The small cubic TiN nitrides are found inside the initial *γ* solid phase, indicating that they have formed in the liquid alloy before solidification. The NbC carbides, containing a certain amount of Ti, are first found near the residual liquid in the sample quenched at 1250 °C. With the decrease of quenching temperature, the size and quantity of NbC increase gradually, while those of TiN do not change significantly. The Laves phase, which is generally considered to be the major microstructural segregation constituent of as-cast IN718 alloy, is observed only near the solute-rich interdendritic liquid of the specimen quenched at 1170 °C. Nb and Mo are enriched in the Laves phase, whereas Al, Fe, Cr, and Ni are depleted.

The observed phase transformations in the microstructures are in agreement with the above interpretations of the DSC profile (Figure 1) and the variation trends of the segregation ratios (Figure 5). A portion of Ti was consumed in the liquid alloy due to the formation of TiN. As the solidification process continued, Ti was discharged from the solidification front to the liquid phase because it is a positive segregation element in the alloy; thus, the content of Ti in the remaining liquid increased again and then precipitated with NbC at around 1250 °C, which causes the SR of Ti to increase first and then decrease. At the same time, the increasing trend of Nb content in the liquid does not seem to be affected by the formation of NbC carbides unless the Laves phase begins to form. Probably because the content of Nb in IN718 alloy is much higher than that of Ti, and the propensity of Nb to segregate to the liquid phase is much stronger than that of Ti. Combining these results with the above conclusions indicates that Nb is the most principal segregation element in IN718 alloy during solidification under slow-cooled conditions, which is abundant in the remaining liquid at a late stage of solidification and involved in promoting the generation and growth of NbC carbides and the Laves phase. Furthermore, since the size and proportion of the Laves phase in as-cast IN718 alloy are much larger compared with NbC [7], and because the Laves phase begins to be generated below 1190 °C, consuming large amounts of Mo and Nb in the remaining liquid, the SRs of Mo and Nb are alleviated in the sample quenched at 1170 °C.

### 3.3. Macrosegregation Prediction

Freckles are the most common macro-segregation defects in nickel-based superalloy castings and ingots. They are featured by the strip-like channels with convective instabilities in the mushy zone during solidification [17]. The convective flow of interdendritic liquid has been explained as the result of the driving force built up due to the density contrast that overcomes the frictional resistance or impedance to the flow; meanwhile, the impedance is related inversely to the permeability of the dendritic array and is directly related to the dynamic viscosity of the liquid [20]. Since the instabilities in the mushy zone are the result of hydrodynamic convection, of the criteria reported in the literature, most researchers choose to use the dimensionless Rayleigh number (Ra) concept as the best suited criterion to predict the onset of freckle formation [21]. The transition point for the solutes’ enriched interdendritic liquid to lose its stability defines the critical number of the freckle formation [18]. Equation (3) is a well-accepted universal Ra to evaluate the freckle formation tendency [10,19]:

(3) $$Ra=g\frac{\Delta \rho \prod }{{\nu f}_{L}R}\sin\alpha \cos(\phi +\alpha )$$

where g is the gravity constant, m·s^−2^; Δ*ρ* is the relative change in liquid density within the mushy zone, kg·m^−3^, using the alloy liquidus as the reference point; Π is the averaged permeability, m^2^; *ν* is the liquid dynamic viscosity, N·s·m^−2^; *f*_L_ is the averaged liquid fraction; R is the crystal growth velocity, m·s^−1^; φ is the angle between the crystal growth and horizontal plane, and α is the angle between the liquid flow direction and the isothermal surface.

The angle parameters and crystal growth velocity can be assumed invariable during solidification in this study due to the same alloy at the same cooling rate [18]. Meanwhile, the discrepancies in viscosity are considered not very significant in the temperature range of mushy zone for superalloys [26]. Accordingly, Equation (3) can be simplified as Equation (4), which was derived by Long et al. [12] from Equation (3), to calculate the relative Ra (Ra_(r)_). Ra_(r)_ focuses on the effects of compositional changes and liquid fraction on the formation of freckles. The larger the Ra_(r)_ value, the greater the tendency for convection and freckles to form. In Equation (4), the driving force for the formation of freckles is the relative change in liquid density, which is mainly affected by the solute redistribution and liquid temperature during solidification. The resistive force is the permeability of the dendritic array, which is mainly influenced by the liquid faction and dendritic structure [12]:

(4) $$Ra_{\left(r\right)}=\frac{\Delta \rho \prod }{f_{L}}$$

The density of liquid nickel-based superalloy is generally considered to be a function of solute concentration and temperature, and can be expressed as [27]:

(5) $$\rho =\sum X_{i}M_{i}/\sum X_{i}V_{i}$$

where *X_i_* is the mole fraction of component *i* in the liquid; *M_i_* is the molecular weight of component *I*; and *V_i_* is the partial molar volume (*V_i_^φ^*) of component *i* present in the alloy, 10^−6^ m^3^·mol^−1^. For solvent component *N_i_*, the partial molar volume is replaced by the molar volume (*V*^0^).

The partial molar volume of the main elements in nickel-based superalloy and the molar volume of solvent component Ni can be calculated by the following equations [27]:

(6) $$V_{\mathrm{Ni}}^{0}(10^{-6}m^{3}\cdot {\mathrm{mol}}^{-1})=7.4287+1.4091\times 10^{-3}(T-1728)$$

(7) $$V_{\mathrm{Cr}}^{\varphi }(10^{-6}m^{3}\cdot {\mathrm{mol}}^{-1})=8.1686+7.7257\times 10^{-4}T$$

(8) $$V_{\mathrm{Al}}^{\varphi }(10^{-6}m^{3}\cdot {\mathrm{mol}}^{-1})=9.3859-2.3468\times 10^{-4}T$$

(9) $$V_{\mathrm{Ti}}^{\varphi }(10^{-6}m^{3}\cdot {\mathrm{mol}}^{-1})=7.8304+1.9697\times 10^{-3}T$$

(10) $$V_{\mathrm{Nb}}^{\varphi }(10^{-6}m^{3}\cdot {\mathrm{mol}}^{-1})=9.2650+9.28\times 10^{-4}T$$

(11) $$V_{\mathrm{Mo}}^{\varphi }(10^{-6}m^{3}\cdot {\mathrm{mol}}^{-1})=11.8445-1.3703\times 10^{-3}T$$

where *T* is the temperature in Kelvin.

**Figure 7:**
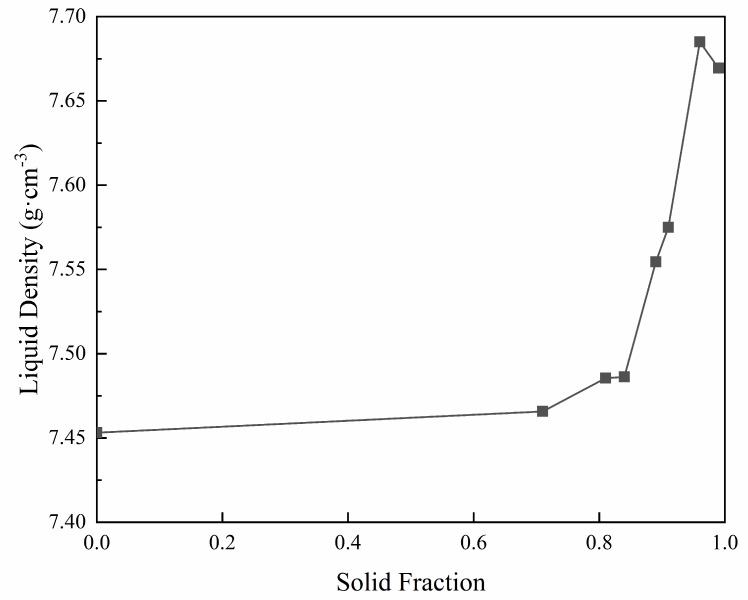
The variation trend of liquid density during solidification.

Based on the above equations and the nominal composition analysis reported in Table 1, the pure liquid density (*ρ*_0_) of the experimental IN718 alloy can be calculated (using the alloy liquidus temperature measured by DSC). Then, combining the equations with the EPMA quantitative measurements, the density of the remaining liquid during solidification at different quenching temperatures (*ρ*_T_) can be obtained, as shown in Figure 7. The liquid density increases with the increase of solid fraction, which indicates that the amount of solutes discharging into the liquid phase increases synchronously. When the solid fraction exceeds 80%, the liquid density increases sharply. However, at the terminal stage of solidification, the propensity of the liquid density to increase reverses slightly, which is most likely due to the fact that the Laves phase begins to generate at this stage, resulting in depletion of the Laves forming elements, such as Nb and Mo, in the residual liquid, as has been analyzed above.

The permeability depends upon the liquid fraction and dendritic structure. Poirier et al. [28] found that there exists a relationship for the permeability, and derived the following expression:

(12) $$\prod =\prod (f_{L})\cdot \prod (d_{1},d_{2})=K_{P}\cdot f_{L}^{a}\cdot d_{1}^{b}\cdot d_{2}^{c}$$

where *d*_1_ and *d*_2_ are the primary and secondary dendrite arm spacing, respectively. *K*_P_, *a*, *b*, and *c* are constants with different values depending on the flow direction. Figure 7 shows the density of the remaining liquid almost increases with decreasing temperature, suggesting that the flow of the interdendritic liquid will be dominated by gravity. In this case, the liquid flow tends to be across the primary dendrite arm rather than along the dendrite arm [28]; thus, the averaged permeability can be calculated by the following equation [18]:

(13) $$\prod =9.66\times 10^{-18}\cdot f_{L}^{3.34}\cdot d_{1}^{0.699}\cdot d_{2}^{2.73}$$

The relative change in the liquid density can be written as:

(14) $$\Delta \rho =\frac{\left|\rho_{T}-\rho_{0}\right|}{\rho_{0}}\times 100\%$$

**Figure 8:**
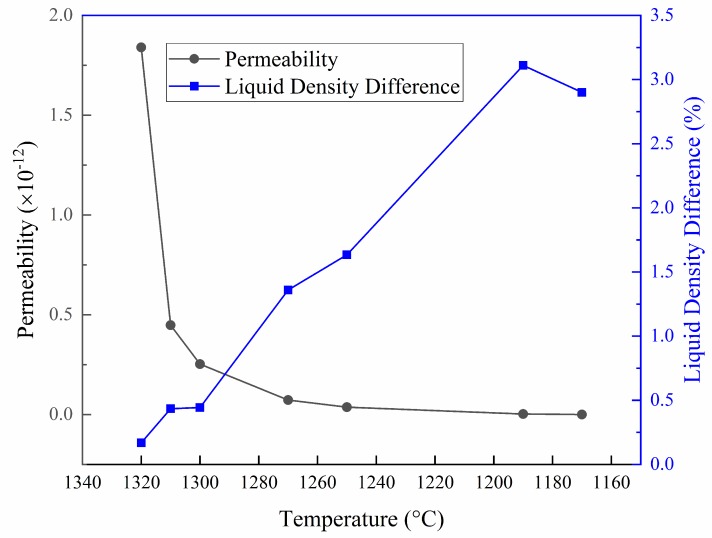
The permeability and the relative liquid density difference during solidification at different temperatures.

According to Equations (13) and (14), the permeability of the dendritic array—the resistive force to form freckles, and the relative liquid density difference—the driving force to form freckles, at each stage of the solidification process could be calculated based on the parameters measured in this study. The results are illustrated in Figure 8, revealing that as the temperature decreases, the relative liquid density difference increases in general, but the permeability continues to decrease. Especially in the initial solidification stage, the permeability shows a sharp downward trend.

**Figure 9:**
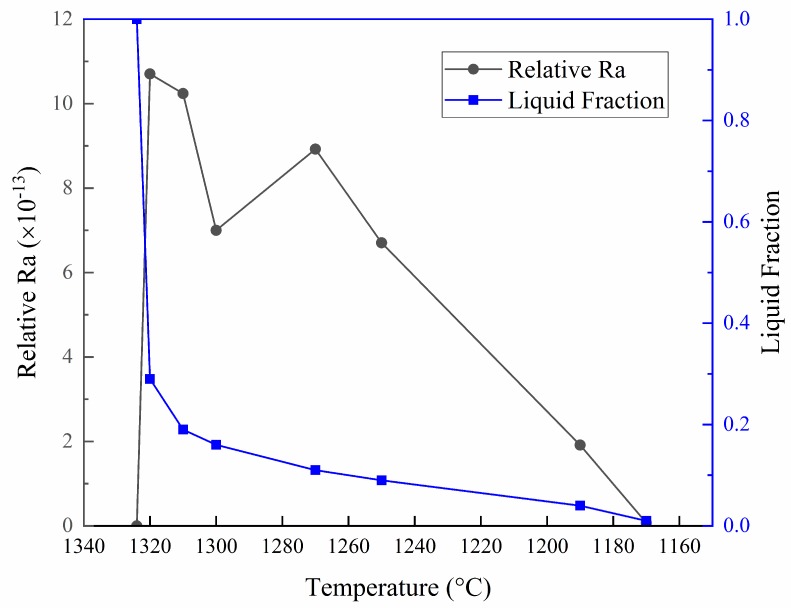
The relative Ra and liquid fraction during solidification at different temperatures.

Inserting Equations (13) and (14) into Equation (4), the characterization of the tendency of the solidification process to develop freckles could be achieved. The calculated Ra_(r)_ and the measured liquid fraction in each sample quenched at different temperatures are superimposed in Figure 9, showing that the maximum value of Ra_(r)_ occurs when the liquid fraction decreases to 0.29, and, at this time, the temperature is 1320 °C. The other peak Ra_(r)_ appears as the temperature decreases to 1270 °C, at which the liquid fraction is approximately 0.1. Thereafter, the Ra_(r)_ decreases gradually in the process of further decreasing the temperature. The bigger the value of Ra_(r)_ is, the higher the freckle formation tendency is. Therefore, the results indicate that when the cooling rate is 5 °C/min, the freckles in as-cast IN718 alloy are most likely to form in the early stage of solidification, at which the liquid fraction in the mushy zone is between 0.3 and 0.2, and the temperature range is about 1320 °C to 1310 °C. Besides, when the temperature decreases to 1270 °C, the propensity of the formation of freckles increases again, and, at this point, the liquid fraction is approximately 0.1. The calculation results are in good agreement with those reported in Reference [12], which analyzed the freckle formation by a semi-experimental approach. It should be noted that the freckles only appear in large-sized superalloy ingots and cannot be detected by nondestructive inspection. However, in the actual production processes of large diameter castings and ingots, it is not possible to determine the specific conditions for the formation of freckles because the quenching verification at high temperatures cannot be achieved. Therefore, this study estimated and predicted the formation trend of freckles by dint of Rayleigh criterion under laboratory conditions. The experimental data may be used to provide a certain reference for industrial productions, and guide the improvement of key procedures for casting and remelting operations.

## 4. Conclusions

A detailed solidification and segregation analysis of as-cast IN718 alloy cooled at a slow cooling rate (5 °C/min) has been performed. The main conclusions were:

1. As the temperature decreases during solidification, the *γ* cell crystal on the solid-liquid interface grows into a regular hexagonal structure, and the intercellular region becomes a continuous reticulation, with small amounts of liquid always existing over a wide temperature range. The variation trend of the solid fraction with solidification time can be well characterized by the Boltzmann distribution and established as *f*s = 1 −/(1 + exp((*t* − 269.5)/55.9)) in this study. The relationship between the transition rate of the solid phase and solidification time exhibits a feature of Gaussian distribution, and its functional form can be expressed as d*f*_s_/d*t* = 1.3 × 10^−4^ + 4.3 × 10^−3^ × exp(−2 × ((*t* − 269.5)/170.2)^2^) in the cooling condition studied here.

2. The degree of segregation of negative segregation elements on the whole follows a slight increasing trend with decreasing temperature in the course of solidification. The order of their degree of segregation is: Fe > Cr > Al. The Ti content in the liquid shows a tendency to increase first and then decrease during solidification. Nb is the most principal positive segregation element under the slow-cooled condition, which shows the strongest tendency to segregate into the liquid. The contents of Nb and Mo in the remaining liquid increase with decreasing temperature before the generation of the Laves phase.

3. The small cubic TiN nitrides are formed in the liquid alloy before solidification. Two major segregation constituents, Laves phase and NbC carbides, are precipitated in the solute-rich residual liquid at different temperatures in the course of solidification. The formation temperature of NbC is about 1250 °C, and the Laves phase is formed in the temperature range of 1190 °C to 1170 °C. The formation of the Laves phase significantly consumes Nb and Mo content in the remaining liquid, resulting in reversal of the trend of increasing liquid density.

4. A quantitative insight on the conditions of freckle formation at a 5 °C/min cooling rate was obtained. The freckles are most likely to form in the early stage of solidification, at which the liquid fraction is between 0.3 and 0.2, and the temperature range is about 1320 °C to 1310 °C. The propensity of the formation of freckles increases again when the temperature decreases to 1270 °C, where the liquid fraction is approximately 0.1.

## Figures and Tables

**Table 1:** Chemical compositions for the experimental IN718 ingot (wt %).

Element	Ni	Cr	Nb	Mo	Al	Ti	C	Si	P	S	Fe
AMS Specification [22]	50.0–55.0	17.0–21.0	4.75–5.50	2.80–3.30	0.20–0.80	0.65–1.15	≤0.08	≤0.35	≤0.015	≤0.015	balance
Measured Results	52.72	18.26	4.92	3.13	0.43	1.13	0.02	0.20	0.01	0.01	19.00

**Table 2:** Measured solid fraction and EPMA results of each quenched sample.

Quenching Temperature (°C)	1320	1310	1300	1270	1250	1190	1170
Solid Fraction	0.71	0.81	0.84	0.89	0.91	0.96	0.99
Average Cr Content in Liquid (wt %)	14.86	15.01	14.89	14.41	14.03	13.88	13.44
Average Fe Content in Liquid (wt %)	11.55	11.40	11.46	11.16	11.35	11.18	10.95
Average Al Content in Liquid (wt %)	0.345	0.355	0.352	0.354	0.341	0.326	0.319
Average Nb Content in Liquid (wt %)	10.44	13.09	14.39	17.60	21.08	24.25	22.21
Average Mo Content in Liquid (wt %)	4.42	4.55	4.90	5.42	5.51	6.22	4.96
Average Ti Content in Liquid (wt %)	1.77	1.84	1.88	2.08	1.88	1.71	1.69

**Table 3:** EDS results from spectrums marked in the FESEM images (wt %).

Elements	N	C	Cr	Fe	Ni	Al	Ti	Nb	Mo
Spectrum 1	10.14	-	0.97	0.78	1.97	5.02	72.07	9.05	-
Spectrum 2	10.78	-	0.86	-	1.20	7.48	69.16	10.53	-
Spectrum 3	-	24.15	3.23	2.37	9.79	-	4.15	53.78	-
Spectrum 4	-	30.52	0.74	-	1.39	-	5.94	61.41	-
Spectrum 5	-	34.55	0.55	-	1.29	-	4.19	59.43	-
Spectrum 6	-	-	13.91	12.14	36.41	0.07	0.81	29.07	6.30
